# IN THEIR OWN WORDS – HOW STUDENT RESPONSES TO OPEN-ENDED QUESTIONS CAN IMPROVE GERONTOLOGY EDUCATION

**DOI:** 10.1093/geroni/igad104.1214

**Published:** 2023-12-21

**Authors:** Nasreen Sadeq

**Affiliations:** University of South Florida, Tampa, Florida, United States

## Abstract

In gerontology education, we know that there are many benefits to assigning students open-ended questions. They allow students to think more critically and can lead to a better understanding of course concepts. Student responses can also help us identify gerontology topics that are not well understood or need reinforcement, as well as topics that can generate interesting in-class discussions. Examining students’ answers can help us fill the gaps in current gerontology education and improve our teaching practices. In this lecture, I will share how I have used content analysis to examine students’ responses from several assignments, and how I have modified lectures and assignments in response. In addition, I will share how I have added some of these assignments to our general education gerontology courses to help students challenge ageist beliefs and acquire an appreciation for gerontology.

